# Optimized workflow of EV enrichment from human plasma samples for downstream mass spectrometry analysis

**DOI:** 10.1007/s12672-024-01248-x

**Published:** 2024-08-27

**Authors:** Patrick Erwied, Yi Gu, Lena Simon, Martin Schneider, Dominic Helm, Maurice Stefan Michel, Philipp Nuhn, Katja Nitschke, Thomas Stefan Worst

**Affiliations:** 1 Department of Urology and Urosurgery, Medical Faculty Mannheim of the University of Heidelberg, Mannheim, Germany; 2 Proteomics Core Facility, German Cancer Research Center (DKFZ), Heidelberg, Germany; 3 Department of Urology, Universitätsklinikum Schleswig-Holstein (UKSH), Campus Kiel, Kiel, Germany

## Abstract

To improve the prognosis of bladder and prostate cancer, highly specific and sensitive biomarkers are needed for early detection, prognosis prediction, and therapeutic stratification. Extracellular vesicles (EV) from plasma could fill this gap due to their potential to serve as cancer biomarkers. However, the enrichment of EV is a major challenge, because the highly abundant plasma proteins are interfering with analytical downstream applications like mass spectrometry (MS). Therefore, the purity requirements of the EV samples must be carefully considered when selecting or developing a suitable EV enrichment method. The aim of this study was to compare a self-designed EV enrichment method based on density cushion centrifugation (DCC) combined with size exclusion chromatography (SEC) and concentration (method 1) with the exoRNeasy midi kit from Qiagen (method 2) and with unprocessed plasma. Furthermore, the single steps of method 1 were evaluated for their effectiveness to enrich EV from plasma. The results showed that the EV samples enriched with method 1 contained the highest levels of EV and exosome markers with simultaneously low levels of highly abundant plasma proteins. In summary, the combination of DCC, SEC and concentration proved to be a promising approach to discover EV-based biomarkers from plasma of cancer patients.

**Supplementary Information:**

The online version contains supplementary material available at 10.1007/s12672-024-01248-x.

## Introduction

More than 50 years ago, nanosized particles were discovered in human blood plasma and termed as platelet dust [1]. As research progressed, different classes of nanoparticles were discovered, and the initial idea of their biological function changed from cellular garbage collection to intercellular communication, making them interesting for biomedical research [1]. To find a generic term, the International Society of Extracellular Vesicles (ISEV) proposes the term extracellular vesicles (EV), to designate cellular particles that are delimited by a lipid bilayer, cannot replicate, and do not contain a functional nucleus [2]. Different subgroups of EV exist and those ranging between 30 and 150 nm in diameter are called exosomes, while those between 100 and 1,000 nm are referred to as microvesicles [3]. Structurally, the phospholipid bilayer of EV is associated with different membrane and transmembrane proteins [4, 5]. Intravesicularly, EV carry various biomolecules like RNA, DNA and proteins, which can be protected from degradation processes by the phospholipid bilayer [6–8]. Both normal cells and tumor cells produce EV, and their presence in various body fluids like breast milk, urine, and blood plasma, renders them promising biomolecules in liquid biopsies [3, 9, 10]. EV have shown potential to serve as biomarkers in different types of diseases like autoimmune diseases, sepsis and neurodegenerative diseases [11, 12]. Considering that cancer is a disease with high incidence and high mortality worldwide, EV are also analyzed as surrogates in applications such as cancer diagnosis, prognosis prediction, and monitoring of oncological therapies [9, 13, 14]. The early detection of cancer is essential to increase the chances for a curative therapy and to improve the prognosis of the further course of the disease [15, 16]. This is particularly true for high-incidence cancers, such as bladder cancer with 573,278 new cases, and for prostate cancer with 1,414,259 new cases worldwide in 2020 [17]. However, due to a lack of minimally invasive, highly specific, and sensitive early detection procedures, cancer such as prostate and bladder cancer are often detected in already progressed stages, which limits the therapeutic options and compromises the prognosis [18, 19]. To overcome this limitation, early detection biomarkers with high specificity and sensitivity are needed [16, 20]. EV might fill this gap as they can carry tumor-associated biomolecules on their surface or as intravesicular cargo [21]. From a clinical and patient perspective, liquid biopsies offer the advantage of being less invasive compared to tissue-based cancer biopsies. This requirement can be fulfilled using plasma, as it is easily accessible and can be obtained under controlled clinical conditions [22]. However, the enrichment of EV from plasma is challenging because plasma is a complex biofluid, containing highly abundant plasma proteins, as well as particles like lipoproteins, which overlap in size and density with EV [23–25]. Additionally, lipoproteins such as very low density lipoprotein particles (VLDL), low density lipoprotein particles (LDL) and high density lipoprotein particles (HDL) exceeded the amount of EV in plasma by at least 10^5^-fold [3, 26]. Different methods for EV enrichment from plasma samples can be found in the literature, with size exclusion chromatography (SEC), density gradient centrifugation (DGC) and kit-based membrane affinity spin column methods being the most frequently used [21, 27–30]. These methods can be used either in combination or individually [27, 29, 30]. The aim of this study was to compare different EV enrichment methods and to identify the most effective method with regard to the downstream analytical application of liquid chromatography tandem mass spectrometry (LC-MS/MS, subsequently abbreviated as MS for better readability) based protoemics. For EV enrichment from human plasma, a self-designed workflow based on density cushion centrifugation (DCC) with subsequent SEC and concentration was established. The single steps as well as the overall method were compared with unprocessed plasma and with EV enriched by using the exoRNeasy midi kit from Qiagen as an example for a ready-to-use approach.

## Materials and methods

### Generation of plasma samples

To generate plasma samples, blood from three healthy volunteers was drawn at the Department of Urology and Urosurgery of the Medial Faculty Mannheim of the University Heidelberg in Germany at two different time points and collected in EDTA tubes. One female and two male donors were included, and the age ranged from 23 to 40 years, with a median age of 31 years. All blood samples were centrifuged at 2,000 × g for 10 min at 4 °C and aliquoted, regardless of further processing. Plasma aliquots were regularly frozen at -80 °C, except for those used for EV enrichment with non-frozen plasma, where the aliquots were used directly.

### Enrichment of EV

**Fig. 1:**
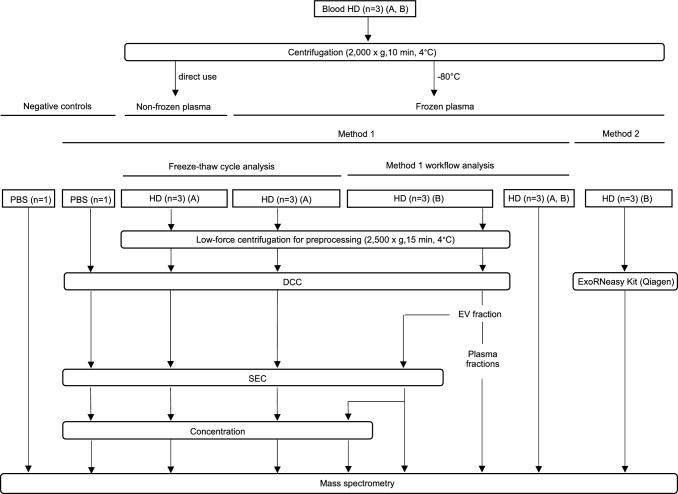
Workflow of sample generation for mass spectrometry (MS). Blood from three healthy donors (HD) and two different dates (**A**, **B**) was used to generate plasma. Samples from left to right: negative controls consisting of unprocessed PBS and PBS after density cushion centrifugation (DCC), size exclusion chromatography (SEC), and concentration. Samples from HD preprocessed with low-force centrifugation, followed by DCC, SEC and concentration (method 1) with non-frozen and frozen plasma for freeze–thaw cycle analysis. HD from frozen plasma for workflow analysis of method 1, after low-force centrifugation and the steps DCC (with subdivision into extracellular vesicles (EV) fraction and plasma fractions); DCC and SEC; DCC, SEC and concentration; as well as unprocessed plasma. HD after EV enrichment with exoRNeasy midi kit (method 2)

For EV enrichment from plasma samples, two different methods were analyzed. Figure 1 gives an overview of the sample preparation for MS analysis.

In the first method, a combination of DCC, SEC and concentration workflow (method 1) was used to enrich EV from plasma (n = 9), after samples were preprocessed by a low-force centrifugation step. To generate negative controls, PBS instead of plasma was used and subjected to the workflow. Additionally, unprocessed PBS was analyzed. Furthermore, samples from unprocessed plasma were generated (n = 6).

For DCC, 3 ml plasma was thawed in water bath at 37 °C and preprocessed by centrifugation at 2,500 × g for 15 min at 4 °C. Additionally, non-frozen plasma was used. The supernatant was mixed with PBS up to a total volume of 15 ml. Open-top thinwall ultra-clear tubes (Beckman Coulter) were filled with 2 ml of 40% Optiprep solution (Serumwerk Bernburg) to generate a density cushion. The plasma-PBS mixture was carefully layered on top of the density cushion. For the DCC, samples were centrifuged in a swing out rotor (SW 32.1 Ti) in an Optima XPN-80 ultra-centrifuge (Beckman Coulter) at 130,000 × g for 16 h at 4 °C [3]. After DCC, approximately 12 ml of liquid was carefully aspirated by pipetting from the top of the tube, to remove lipoproteins and plasma fractions. The EV enriched fraction was collected directly above the density cushion in 1 ml volume and transferred into a reaction tube. After equilibration and washing of the SEC column at room temperature (RT), the EV enriched DCC fraction was loaded onto the SEC column (Izon Science, qEV ORIGINAL Gen2, 35 nm). EV were eluted with PBS and the EV enriched SEC fractions were concentrated in a centrifugal filter unit at 4,000 × g for 17 min at RT (Amicon Ultra-2 centrifugal filter, 10 kDa, Merck) [31]. EV were recovered by centrifuging the inverted unit at 1,000 × g for 2 min at RT [32].

In method 2, EV enrichment was performed using a commercially available column-based kit (exoRNeasy midi kit, Qiagen). 2 ml plasma from healthy donors (n = 3) were thawed in a water bath at 37 °C and centrifuged at 16,000 × g for 30 min at 4 °C. The supernatant was sterile filtered (0.2 µm) and mixed with 1 volume of XBP buffer. The plasma-XBP mixture was transferred onto the exoEasy membrane affinity column and centrifuged at 500 × g for 1 min at RT. The column was washed with 3.5 ml XWP buffer and centrifuged at 4,700 × g for 5 min at RT. EV were eluted in 100 µl XE buffer [33].

#### Sample generation for analysis of EV enrichment workflow

To analyze the entire EV enrichment workflow of method 1, plasma was completely or partially subjected to the above-described workflow. The following samples were generated:

- frozen plasma as unprocessed samples (n = 6 from three healthy donors at two dates)
- frozen plasma subjected to low-force centrifugation and single step DCC (n = 3) to collect 7 plasma fractions of 2 ml each and 1 EV enriched fraction of approximately 1 ml. For MS analysis, sample volumes of 20.3 to 23.6 µl were taken from the EV fractions, and the remaining volume was further processed
- EV enriched fraction of approximately 1 ml from frozen plasma after low-force centrifugation and DCC subjected to SEC (n = 3). Samples of 36 µl for MS analysis were taken from the filter units, and the remaining volume was further processed
- EV enriched fraction of approximately 1 ml from frozen plasma after low-force centrifugation and DCC and SEC subjected to concentration (n = 3). Sample volumes of 20.3 to 31.2 µl were taken for MS analysis, and the remaining volume was further processed
- frozen plasma subjected to low-force centrifugation and DCC followed by SEC and concentration (n = 3)
- non-frozen plasma subjected to low-force centrifugation and DCC followed by SEC and concentration (n = 3)

To assess the impact of the freeze–thaw cycle, blood from the same collection date was used to generate non-frozen and frozen plasma samples.

#### Nanoparticle tracking analysis (NTA), flow cytometry and protein concentration

The particle concentrations and the median particle diameters were measured by nanoparticle tracking analysis (NTA) using the Zetaview device (Particle Metrix) at 488 nm laser wavelength in scatter mode. The camera parameters were set to 80 for sensitivity, 100 for shutter and 30 for frame rate. The post-acquisition parameters were set to 30 for minimal brightness, 1000 for maximal area, 10 for minimal area and 15 for trace length. For quality control, flow cytometry analysis was performed. For this purpose, particle amounts of 9 × 10^8^ to 1 × 10^10^ EV were coupled to magnetic beads (EV-Human CD9 Flow Detection Reagent, Invitrogen) and incubated overnight at 4 °C. A blocking step was performed with FcR antibody (Miltenyi Biotec) for 15 min at RT. After washing the beads with 0.1% BSA/PBS, the primary antibodies against CD9 (clone M-L13, PE-conjugated), CD63 (clone H5C6, PE-Cy7-conjugated), and CD81 (clone JS-81, FITC-conjugated; all three from BD Biosciences) were added. The mixture was incubated for 30 min at RT and washed three times. Furthermore, a negative control was used, which was not stained with the CD9, CD63, or CD81 antibodies. The flow cytometry analysis was performed with Cytek Northern Lights in the FlowCore Mannheim of the Medical Faculty Mannheim of the University Heidelberg and analyzed with FlowJo 10 Software. To determine the protein concentration, EV were lysed for 5 min at RT in 0.4% SDS. After an incubation for 15 min with the Qubit working solution, EV were measured with a final concentration of 0.01% SDS in the Qubit-Protein Assay (Thermo Fisher Scientific) using the Qubit 3 Fluorometer. To compare the effectiveness of the partial and the complete workflow of method 1 with method 2 and with unprocessed plasma, the particle and protein amounts enriched from 1 ml plasma were calculated by considering the sample volumes, protein concentrations, particle concentrations, and the initial plasma volumes.

#### Sample preparation and LC–MS/MS analysis

For LC-MS/MS, samples were adjusted with PBS to a protein concentration of 10 µg in 36 µl. For EV lysis, 4 µl of RIPA buffer were added and samples were frozen at -80 °C. Due to low protein concentrations in some samples after DCC and SEC without concentration, 36 µl were used (protein amounts of 3.1 µg to 3.8 µg in 40 µl). MS of block-randomized samples was performed at the Proteomics Core Facility at the German Cancer Research Center (DKFZ) in Heidelberg. Hereby, the single-pot, solid-phase-enhanced sample-preparation (SP3) technology was used to prepare the samples for MS [34]. The proteins were digested with Trypsin using an AssayMAP Bravo liquid handling system (Agilent technologies) running the autoSP3 protocol [34].

A liquid chromatography-MS/MS (LC–MS/MS) analysis was carried out on an Ultimate 3000 Ultra-high-performance liquid chromatography system (UPLC) (Thermo Fisher Scientific), directly connected to an Orbitrap Exploris 480 mass spectrometer for a total of 60 min. Peptides were online desalted on a trapping cartridge (Acclaim PepMap300 C18, 5 µm, 300 Å wide pore; Thermo Fisher Scientific) for 3 min using 30 µl/min flow of 0.05% trifluoroacetic acid (TFA) in water. The analytical multistep gradient (300 nl/min) was performed using a nanoEase MZ Peptide analytical column (300 Å, 1.7 µm, 75 µm × 200 mm; Waters) using solvent A (0.1% formic acid in water) and solvent B (0.1% formic acid in acetonitrile). For 43 min the concentration of solvent B was linearly ramped from 5 to 30%, followed by a quick ramp to 78%. After two minutes the concentration of solvent B was lowered to 2% and a 10 min equilibration step appended. Eluting peptides were analyzed in the mass spectrometer using data independent acquisition (DIA) mode. A full scan at 120,000-fold resolution (380–1400 m/z, 300% automatic gain control (AGC) target, 45 ms maximum inject time (maxIT)) was followed by 20 quadrupole isolation windows (400–1,000 m/z; 1 Dalton (Da) overlap, variable width). For the second stage of mass analysis (MS2) scans, the AGC target was set to 1.000%, 30,000-fold resolution, maxIT 54 ms, and collision energy of 28%).

#### Data analysis

Analysis of RAW files was performed with Spectronaut (version 17.1.221229.55965; Biognosys) in directDIA + (deep) library-free mode. Default settings were applied with the following adaptions: Within the *Pulsar Search* in *Peptides* the *Max Peptide Length* was set to 35, in *Result Filters* the *Peptide Charge* was enabled and the *Max Charge* set to 6 and the *Min Charge* set to 2. Within DIA Analysis under *Identification* the *Precursor PEP Cutoff* was set to 0.01, the *Protein Qvalue Cutoff (Run)* set to 0.01 and the *Protein PEP Cutoff* set to 0.05. In *Quantification* the *Proteotypicity Filter* was set to *Only Protein Group Specific*, the *Cross-Run Normalization* was disabled, the *Quantification window* was set to *Not Synchronized* and the *Major Group Quantity* was set to *Sum peptide quantity*. The data was searched against the human proteome from Uniprot (human reference database with one protein sequence per gene, containing 20,591 unique entries from 01.03.2023) and the contaminants FASTA from MaxQuant (246 unique entries from 22.12.2022).

The MS data, which contained annotated proteins and label-free quantification intensities, were analyzed using RStudio (R programming language, version 4.3.1). To create principal component analysis (PCA) and heatmaps, log2 values of protein intensities were used. To generate Venn diagrams, PBS after DCC, SEC and concentration (n = 1) was used as negative control and subtracted from detected proteins. In case of unprocessed plasma, the negative control used for subtraction was unprocessed PBS (n = 1).

## Results

### Median diameters, particle and protein amounts from 1 ml plasma, and the expression of tetraspanins depended on EV enrichment levels

First, a quality control of samples resulting from different EV enrichment methods, EV enrichment steps and from unprocessed plasma was preformed (Fig. 1). Table 1 shows the median particle diameters, the calculated particle and protein amounts enriched from 1 ml plasma, as well as the protein amounts and sample volumes used for MS analysis.

**Table 1:** Median particle diameters resulting from different extracellular vesicle (EV) enrichment steps of method 1, from different EV enrichment methods, and from unprocessed plasma

EV enrichment step	Median particle diameter [nm]	Particles from 1 ml plasma [particles]	Protein from 1 ml plasma [µg]	MS Protein amount [µg]	MS sample volume [µl]
DCC	145.9–156.7	4.3 × 10^9^–6.7 × 10^9^	14,133.3–16,400.0	10.0	20.3–23.6
DCC and SEC	154.1–169.1	2.1 × 10^9^–3.1 × 10^9^	57.7–70.0	3.1–3.8	36
DCC, SEC and concentration (frozen plasma)	148.1–165.8	7.5 × 10^8^–1.8 × 10^9^	12.9–25.9	10.0	20.3–31.2
DCC, SEC and concentration (non-frozen plasma)	157.5–173.4	6.4 × 10^8^–8.6 × 10^8^	12.2–21.6	10.0	21.4–29.2
exoRNeasy midi kit	171.3–189.2	2.1 × 10^9^–2.6 × 10^9^	50.4–51.5	10.0	9.7–9.9
Unprocessed	100.2–130.6	1.9 × 10^11^–2.3 × 10^12^	65,600.0–86,000.0	10.0	5.5–24.4

Particle and protein amounts enriched from 1 ml plasma were calculated by considering sample volumes, protein concentrations, particle concentrations, and the initial plasma volume. Summary of protein amounts and sample volumes of MS samples

The lowest EV diameters were observed for unprocessed plasma, ranging from 100.2 nm to 130.6 nm. Samples generated from frozen and non-frozen plasma after DCC, SEC and concentration showed diameters between 148.1 to 173.4 nm. In the case of the single steps from method 1, DCC, and DCC followed by SEC, diameters ranged from 145.9 to 169.1 nm. For the exoRNeasy midi kit, the diameters were between 171.3 to 189.2 nm.

The particle and protein amounts from 1 ml plasma were lowest in samples from frozen and non-frozen plasma after DCC, SEC and concentration, with values ranging from 6.4 × 10^8^ to 1.8 × 10^9^ particles, and with 12.2 to 25.9 µg protein. This was followed by the exoRNeasy midi kit, with ranges from 2.1 × 10^9^ to 2.6 × 10^9^ particles, and with 50.4 to 51.1 µg protein. DCC with SEC but without concentration resulted in 2.1 × 10^9^ to 3.1 × 10^9^ particles, and 57.7 to 70.0 µg protein. Single step DCC showed high values of 4.3 × 10^9^ to 6.7 × 10^9^ particles, and 14,133.3 to 16,400.0 µg protein, which was only exceeded by unprocessed plasma with 1.9 × 10^11^ to 2.3 × 10^12^ particles and 65,600.0 to 86,000.0 µg protein.

**Fig. 2:**
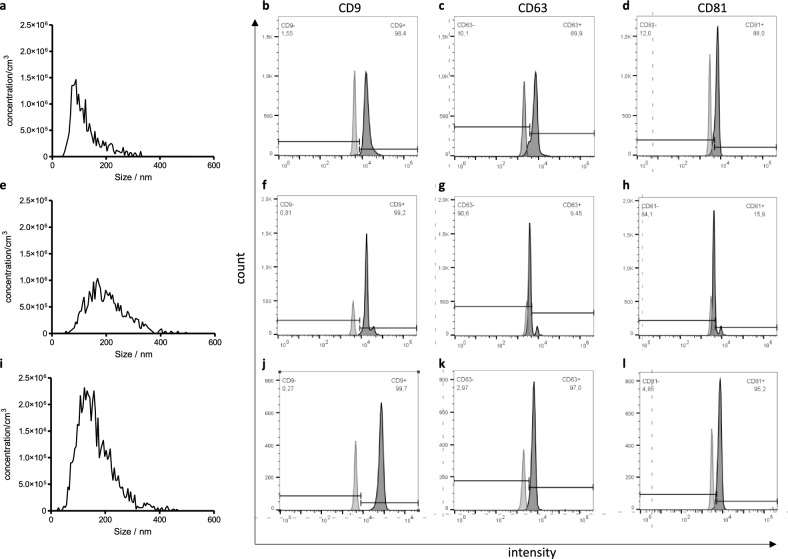
Characterization of EV from unprocessed plasma (**a**–**d**), the exoRNeasy midi kit (**e**–**h**) as well as DCC, SEC and concentration (**i**-l) using NTA and flow cytometry from one healthy donor. In flow cytometry analysis, CD9 was detectable in all EV samples, whereas CD63 and CD81 were only detectable on EV from unprocessed plasma and after EV enrichment with DCC, SEC and concentration (light grey: unstained control; dark grey: stained sample)

For the single plasma fractions after DCC, the particle and protein amounts enriched from 1 ml plasma resulted in a calculated range of 6,5 × 10^9^ to 3,5 × 10^11^ particles, with protein amounts ranging from 2,560.0 to 11,200.0 µg. The median diameters were within a range of 90.2 to 109.3 nm (Supplementary Fig. 1 a, b). Furthermore, a typical EV distribution determined by NTA was observed and is shown in Fig. 2.

In addition, the EV from unprocessed plasma, EV enriched with the exoRNeasy midi kit as well as EV enriched with DCC, SEC and concentration were analyzed by flow cytometry. The typical EV surface marker CD9, CD63 and CD81 were detectable in unprocessed plasma and in EV enriched with the combination method DCC, SEC and concentration, whereas the detection of CD63 and CD81 was not possible on the EV after enrichment with exoRNeasy midi kit (Fig. 2).

### Principal Component Analysis (PCA) showed differences between EV enrichment levels

**Fig. 3:**
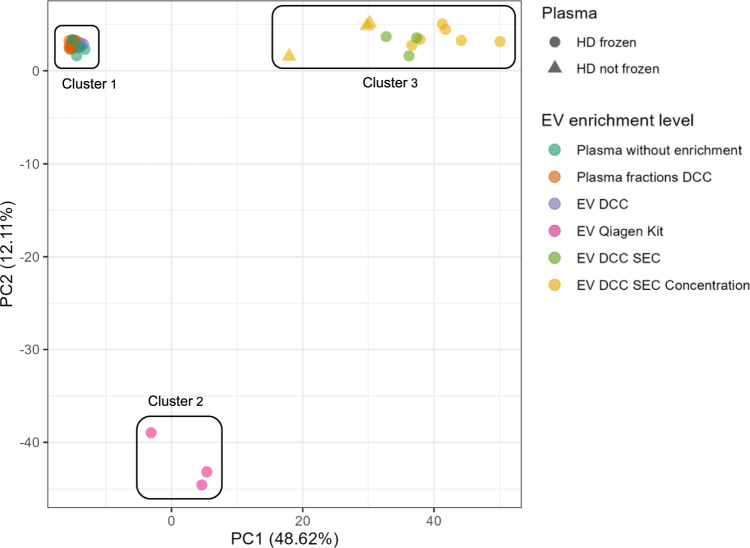
Principal Component Analysis (PCA) of samples generated with frozen and non-frozen plasma from HD. Sample size was as follows: Plasma without enrichment (n = 6), Plasma fractions after DCC (n = 21, 7 samples of 3 donors each), EV after DCC (n = 3), EV after exoRNeasy midi kit (n = 3), EV after DCC, SEC (n = 3), EV from frozen plasma after DCC, SEC and concentration (n = 6), EV from non-frozen plasma after DCC, SEC and concentration (n = 3). The proportion of the total variance within the data is represented by the values of PC1 (48.62%) and PC2 (12.11%)

To assess the influence of different EV enrichment levels, a PCA was performed, which included all sample groups (Fig. 3).

The principal component 1 (PC 1) explains 48.62% of the data variance, while PC 2 accounts for 12.11%. In total, three clusters could be identified. The first cluster was dense and consisted of plasma without enrichment, plasma fractions after DCC and EV enriched fractions after DCC (upper left corner). The second cluster included EV enriched with the exoRNeasy midi kit (lower left corner). The third cluster consisted of EV enriched by DCC and SEC with and without further concentration (upper right corner). Furthermore, this cluster contained EV enriched from frozen and non-frozen plasma, with one sample from non-frozen plasma located further away.

### Heatmap revealed highest detection of EV and exosome markers for method 1

**Fig. 4:**
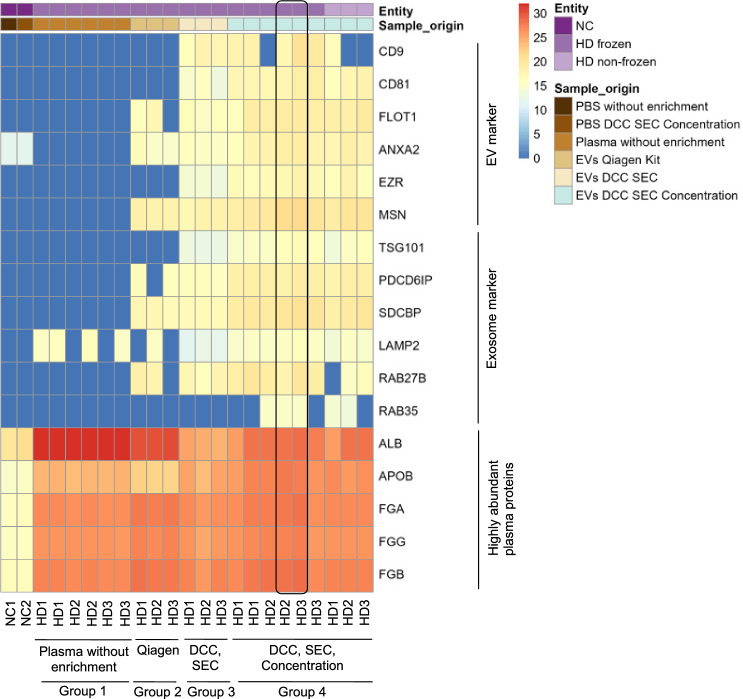
Heatmap of MS samples from HD with different EV enrichment levels, subdivided into four distinct groups. From left to right: negative controls (NC) consisting of unprocessed PBS and PBS after DCC, SEC and concentration with overall low marker detection. Groups are represented at the bottom of the figure. Group 1: Plasma without enrichment (n = 6) showed low EV and exosome marker detection. Group 2: EV enriched with Qiagen exoRNeasy midi kit (n = 3) showed middle EV and exosome marker detection. Group 3: EV after DCC and SEC (n = 3) showed high EV and exosome marker detection. Group 4: EV after DCC, SEC and concentration (n = 9) showed the highest EV and exosome marker detection, regardless of whether the plasma was frozen or non-frozen. Box highlighting two samples from group 4 in which all EV and exosome markers were detected. Color coding for detected protein intensities: zero to low in blue; low to middle in yellow; middle to high in red

To assess the quality of samples with different EV enrichment steps, a heatmap based on protein group intensity values was created (Fig. 4).

The heatmap shows the gene names of specific proteins, which are described in the literature as either EV markers or exosome markers or highly abundant plasma proteins [5, 25, 35–38]. These were namely Cluster of Differentiation 9 (CD9), CD81, Flotillin-1 (FLOT1), Annexin A2 (ANXA2), Ezrin (EZR) and Moesin (MSN) as EV markers, Tumor Susceptibility Gene 101 (TSG101), Programmed Cell Death 6-Interacting Protein (PDCD6IP), Syntenin-1 (SDCBP), Lysosome-associated membrane glycoprotein 2 (LAMP2), Ras-related protein Rab-27B (RAB27B) and Ras-related protein Rab-35 (RAB35) as exosome markers. Albumin (ALB), Apolipoprotein B-100 and B-48 (APOB), Fibrinogen alpha chain (FGA), Fibrinogen gamma chain (FGG) and Fibrinogen beta chain (FGB) are shown as highly abundant plasma proteins [5, 25, 35–38].

The heatmap shows that in unprocessed plasma (group 1), only the exosome marker LAMP2 was detected with a simultaneously high level of highly abundant plasma proteins. The negative controls showed a very similar detection of highly abundant plasma proteins and EV marker ANXA2, regardless of whether the PBS was unprocessed or processed by using method 1. Compared to all other samples, the protein levels in the negative controls were the lowest. When using the exoRNeasy midi kit (group 2), the EV and exosome marker detection was in the middle range. After EV enrichment with DCC and SEC but without concentration (group 3), high levels of EV and exosome markers were detected, while highly abundant plasma proteins were at their lowest. In samples after DCC, SEC and concentration (group 4), all EV and exosome markers were detected in 2 out of 9 samples (22%), while 1 or 2 markers were missing in 7 samples (78%). The amount of highly abundant plasma proteins ranged between those of group 2 and 3. No major differences between marker intensities were observed between frozen and non-frozen plasma. The comparison of samples after DCC and SEC with and without concentration showed that the omission of the concentration step resulted in slightly lower marker intensities overall (group 3).

### Venn diagrams showed the highest number of exclusive proteins for method 1

**Fig. 5:**
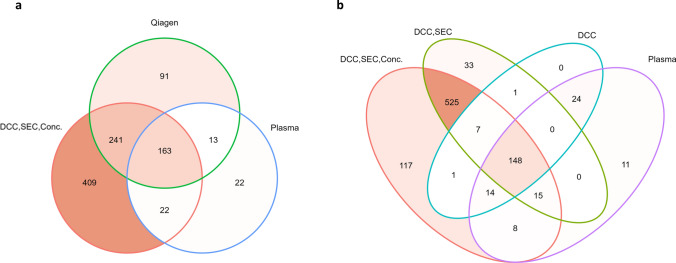
**a** Three-set Venn diagram comparing different EV enrichment methods with unprocessed plasma. Samples from HD after EV enrichment with DCC, SEC and concentration (DCC,SEC,Conc.) (each 3 ml plasma), and with Qiagen exoRNeasy kit (Qiagen) (each 2 ml plasma), as well as unprocessed plasma (Plasma) (10 µg protein). Detected EV proteins, after subtraction of PBS control subjected to DCC, SEC and concentration (n = 1) or subtraction of unprocessed PBS control (n = 1) in case of unprocessed plasma. **b** Four-set Venn diagram comparing different EV enrichment steps with unprocessed plasma. From left to right: samples from HD after EV enrichment with DCC, SEC and concentration, with DCC and SEC as well as with single step DCC, and unprocessed plasma

Venn diagrams were created to visualize the number of identified proteins in relation to the different EV enrichment steps (Fig. 5a).

EV enriched with DCC, SEC and concentration (n = 6) yielded the highest values with a total of 835 proteins and 409 exclusive proteins. This was followed by the exoRNeasy midi kit (n = 3), which yielded 508 proteins and 91 exclusive proteins, while unprocessed plasma (n = 6) resulted in 220 proteins and 22 exclusive proteins.

The comparison of different EV enrichment steps with unprocessed plasma, showed that 117 proteins could be identified exclusively after DCC, SEC and concentration (n = 6) (Fig. 5 b), while the total number was 835. Only 33 proteins were identified exclusively after DCC and SEC without concentration (n = 3) with 729 proteins in total. Single step DCC (n = 3) did not result in any exclusively identified proteins compared to the other samples, albeit 195 proteins were identified. In unprocessed plasma (n = 6) only 11 proteins with a total of 220 were found.

## Discussion

In the literature, various methods exist for EV enrichment, such as ultracentrifugation (UC), SEC, Asymmetric Flow Field Fractionation (AF4) which is based on a cross-flow filtration principle, microfluidic devices based on different physical or mechanical EV properties such as ExoChip, as well as ultrafiltration, immunoaffinity, membrane affinity-based methods such as exoRNeasy, density gradient centrifugation, and precipitation-based methods like ExoQuick [3, 38–41]. These methods can be applied separately or in combination [3, 27, 30, 38, 42]. The choice of a separate method or a combination depends on the scientific question, the intended downstream analysis, and the amount of starting material [3, 27, 30, 41]. To obtain EV samples that contain only a small proportion of highly abundant plasma components such as lipoproteins, a combination of methods based on different physical principles, such as particle size and particle density, can be applied [27, 29]. In the literature the method combination of UC and SEC is described as an effective method to enrich EV from plasma and serum samples with a simultaneously low amount of highly abundant proteins and lipoproteins and is therefore suitable for subsequent MS analysis [39, 43]. In principle, our approach described in this study is a combination of UC and SEC. Since some types of lipoproteins, which are very abundant, elute from SEC columns in the same size range as EV, as many lipoproteins as possible were separated in a first EV enrichment step by applying DCC, a variant of UC.

In this study a combination of DCC, SEC and concentration resulted in a higher detection of total proteins as well as EV and exosome markers compared to unprocessed plasma and EV enriched with the exoRNeasy midi kit. In accordance with the literature, these results suggest that the purity of the EV samples is a key factor when applying MS analysis [27, 37, 44]. Highly abundant plasma proteins like albumin can mask the detection of low abundant EV proteins in MS, such as tumor-associated proteins [38, 45]. This explains, why in unprocessed plasma, in EV enriched fractions and in plasma fractions after single step DCC (Supplementary Fig. 2), only one exosome marker could be detected. The EV enrichment with the exoRNeasy midi kit resulted in the second highest albumin levels, which is associated with a lack of the EV markers CD9 and CD81 and the exosome markers EZR, TSG101 and RAB35 in all samples. Similar results were observed in the study of Stranska et al., in which the proteins CD81 and TSG101 were not detectable in western blots after EV enrichment with exoEasy columns [28, 33]. Furthermore, the authors concluded that the use of exoEasy columns resulted in samples consisting of a heterogeneous mixture of plasma particles including EV, proteins, and lipoproteins [28]. The samples after DCC and SEC without concentration showed slightly less markers of highly abundant plasma proteins, EV and exosomes compared to the concentrated samples. This is because the SEC process dilutes the EV sample, resulting in lower detection levels of the markers [46]. Thus, volume reduction by concentration resulted in higher detection of EV and exosome markers, qualifying this step as necessary for EV enrichment. The detection of partly all EV and exosome markers in the samples after DCC, SEC and concentration (full workflow of method 1), emphasizes the effectiveness of our method for the enrichment of EV from plasma with simultaneously low levels of highly abundant plasma proteins. The general influence of the EV enrichment level on the sample characteristics can be derived from the three clusters shown in Fig. 3. An EV enrichment with DCC, SEC and concentration yielded a clearly separated cluster compared to the others. The effectiveness of method 1 was additionally confirmed by EV characterization using NTA measurements and flow cytometry. The low particle and protein amounts of method 1 indicate effective depletion of highly abundant plasma proteins and lipoproteins compared to unprocessed plasma and the exoRNeasy kit (Supplementary Fig. 3a, b) [28, 30, 47]. Additionally, the small median particle diameters and the high particle and protein amounts in unprocessed plasma were caused by the presence of a high proportion of small lipoprotein particles, such as chylomicrons, in a size range of 75 to 1,200 nm (Supplementary Fig. 3c) [3, 30]. Regarding the single plasma fractions after DCC, the high particle amounts, especially in the first milliliters, indicate a high content of lipoproteins in these fractions (Supplementary Fig. 1a). This observation confirms the effectiveness of the DCC step to remove lipoproteins from the EV fraction located above the density cushion [42, 48]. The effect of a freeze–thaw cycle can seemingly be neglected, as only minor differences in EV and exosome marker detection were observed when comparing samples generated from non-frozen and frozen plasma. The negative controls showed a low level of contamination with highly abundant plasma proteins and with an EV marker, which may have several explanations. One possibility could lie in MS-based proteomic analysis. The measurements of the different conditions have been performed in a block-randomized manner, which is a standard operation procedure in LC–MS based analysis to avoid any acquisition bias [49]. Although extensive washing was carried out after each sample, a carry-over, especially of highly abundant peptides from a previous sample, cannot be completely excluded. This becomes even more apparent if the sample is a blank negative control, while a sample with regular signal intensity might not be affected. Taken together it can be concluded that the sample purity and the annotation of proteins are positively correlated, as already described in the literature [37, 44].

In summary, the combination of DCC, SEC and concentration for EV enrichment from plasma proved to be more effective than the other conditions (unprocessed plasma, single step DCC, the exoRNeasy midi kit and DCC and SEC without concentration) with regard to subsequent MS-based proteomic analysis. By using this method, the highest levels of EV and exosome markers were detected, and the largest number of unique proteins could be identified. Therefore, the combination of DCC, SEC and concentration appears to be a promising option for biomarker discovery studies from plasma of cancer patients.

### Supplementary Information

Additional file 1

## Data Availability

Data is provided within the manuscript or supplementary information files.
