# Supplementary material for: Optimized workflow of EV enrichment from human plasma samples for downstream mass spectrometry analysis
Source: Discov Oncol. 2024 Aug 27;15:374. doi: 10.1007/s12672-024-01248-x (PMC11349724; doi:10.1007/s12672-024-01248-x)
Supplement: Supplementary file 1 — Additional file 1 [file 12672_2024_1248_MOESM1_ESM.pdf]

Supplementary material

Article type: Analysis

Journal name: Discover Oncology

Autors: Patrick Erwied, Yi Gu, Lena Simon, Martin Schneider, Dominic Helm, Maurice Stefan Michel, Philipp Nuhn, Katja Nitschke, Thomas Stefan Worst

Corresponding author

Affiliation: Department of Urology and Urosurgery, Medical Faculty Mannheim of the University of Heidelberg, Mannheim, Germany.

E-Mail address: thomas.worst@medma.uni-heidelberg.de

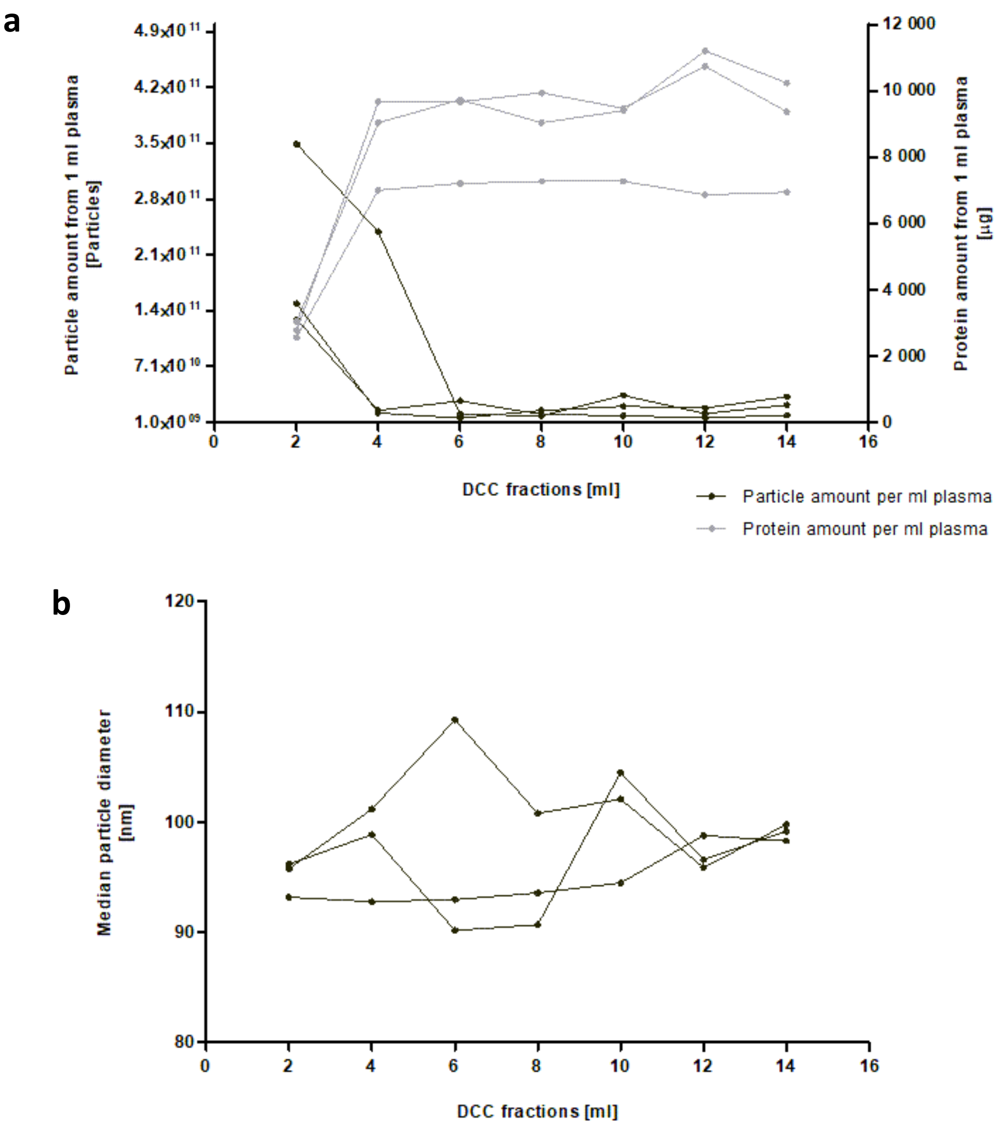

Supplementary Fig 1 Characterization of plasma fractions after single step DCC (n = 3). Particle amounts and protein amounts (a). Median particle diameters (b).

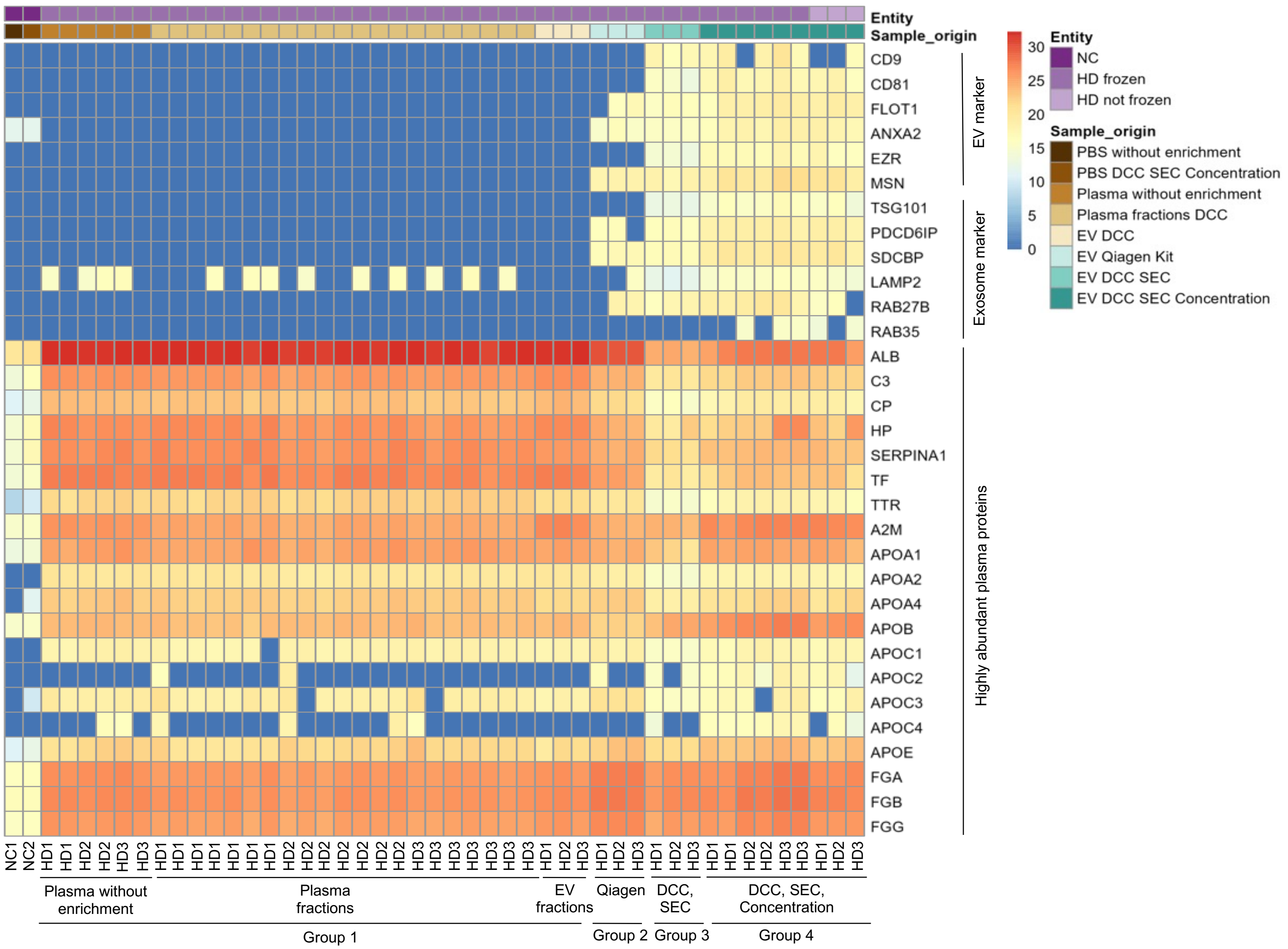

Supplementary Fig 2 Heatmap of MS samples from HD with different EV enrichment levels, subdivided into four distinct groups. From left to right: negative controls (NC) consisting of unprocessed PBS and PBS after DCC, SEC and concentration with overall low marker detection. Groups are represented at the bottom of the figure. Group 1: Plasma without enrichment (n = 6), plasma fractions after DCC (n = 21) and EV enriched fractions after DCC (n = 3), showed low EV and exosome marker detection. Group 2: EV enriched with Qiagen exoRNeasy kit showed middle EV and exosome marker detection. Group 3: EV after DCC and SEC showed high EV and exosome marker detection. Group 4: EV after DCC, SEC and concentration showed the highest EV and exosome marker detection, regardless of whether the plasma was frozen or not frozen. Color coding for detected protein intensities: zero to low in blue; low to middle in yellow; middle to high in red.

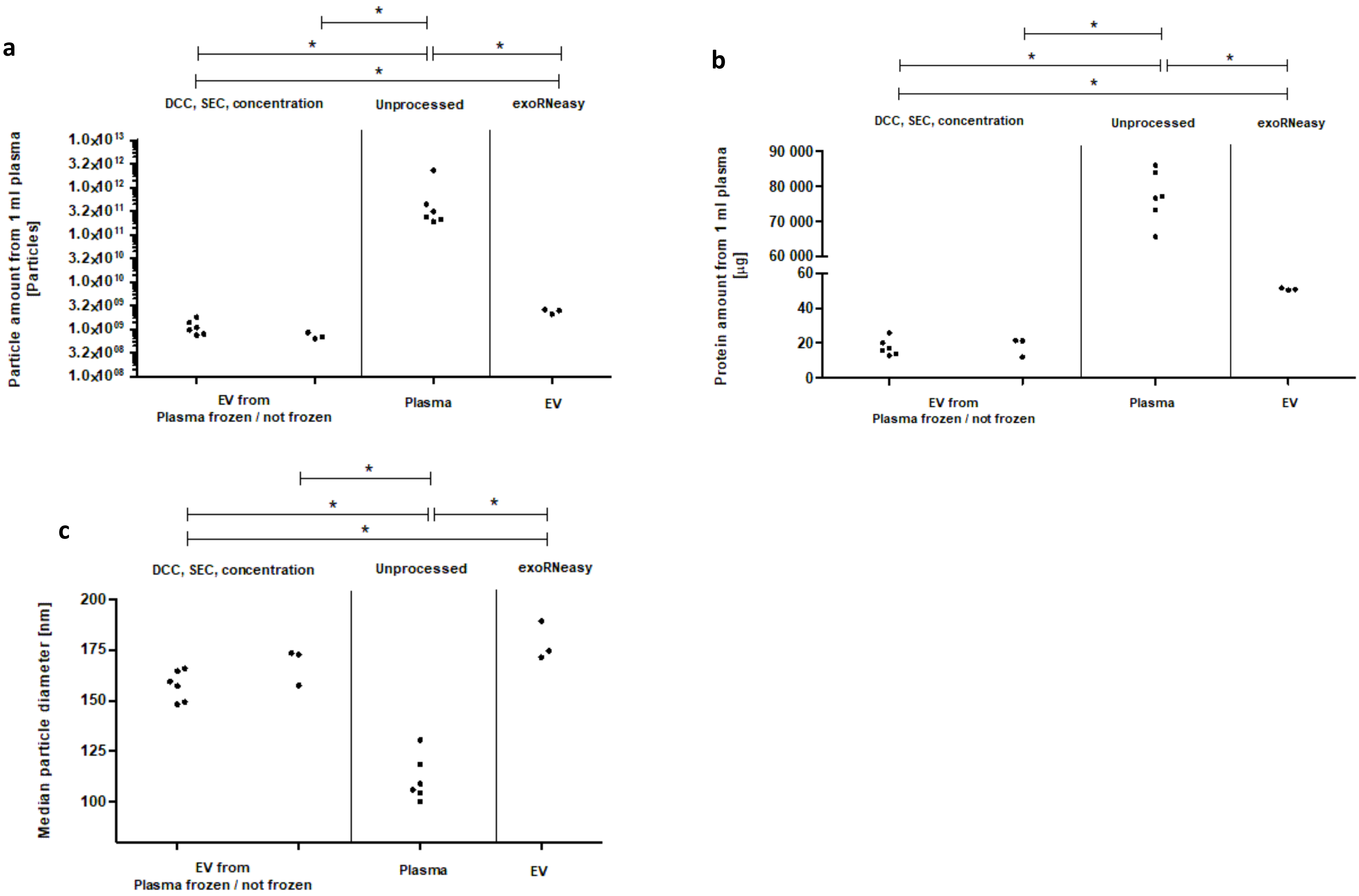

Supplementary Fig 3 Particle amounts (a), protein amounts (b), and median particle diameters (c) of samples generated from plasma of HD by different EV enrichment methods. Left: EV enriched from frozen plasma (n = 6) and non-frozen plasma (n = 3) by combination of DCC, SEC and concentration. Middle: Unprocessed plasma (n = 6). Right: EV enriched with exoRNeasy kit (Qiagen) (n = 3). Statistics: Kruskal-Wallis test with post-hoc pairwise Wilcoxon rank-sum tests with Benjamini-Hochberg adjustment for multiple comparisons using R.; p-value ≤ 0.05 (*).
